# Estimating the survival benefits gained from providing national cancer genetic services to women with a family history of breast cancer

**DOI:** 10.1038/sj.bjc.6601794

**Published:** 2004-04-20

**Authors:** G L Griffith, R T Edwards, J Gray, C Wilkinson, J Turner, B France, P Bennett

**Affiliations:** 1 Centre for the Economics of Health, Institute of Medical and Social Care Research, University of Wales, Bangor, Gwynedd LL57 2UW, UK; 2 Institute of Medical Genetics, Cardiff and Vale NHS Trust, University Hospital of Wales, Heath Park, Cardiff CF14 4XW, UK; 3 Department of General Practice, North Wales Section, University of Wales College of Medicine, Wrexham Technology Park, Wrexham LL13 7YP, UK; 4 Department of Psychology, University of Wales, Swansea, Singleton Park Swansea SA2 8PP, UK

**Keywords:** breast cancer, Markov modelling, survival, BRCA1/2

## Abstract

The aim of this paper is to compare a service offering genetic testing and presymptomatic surveillance to women at increased risk of developing breast cancer with its predecessor of no service at all in terms of survival and quality-adjusted survival (QALYs) by means of a Markov cohort chain simulation model. Genetic assessment and presymptomatic care provided between 0.07 – 1.61 mean additional life years and 0.05 – 1.67 mean QALYs over no services. Prophylactic surgery and surveillance extended mean life expectancy by 0.41 – 1.61 and 0.32 – 0.99 years, respectively over no services for high-risk women. Model outcomes were sensitive to all the parameters varied in the sensitivity analysis. Providing cancer genetic services increase survival and as long as services do not induce adverse psychological effects they also provide more QALYs. The greatest survival and QALY benefits were found for women with identified mutations. As more cancer genes are identified, the survival and cost-effectiveness of genetic services will improve. Although mastectomy provided most additional life years, when quality of life was accounted for oophorectomy was the optimal strategy. Delayed entry into coordinated genetic services was found to diminish the average survival and QALY gains for a woman utilising these services.

Despite the recognition by clinicians for over a century that a hereditary predisposition to cancer exists in certain families, the possibility of utilising this information to help these patients has only become available in the 1990s with the discovery of cancer susceptibility genes (Steel *et al*, 1999). Two of the first common cancer genes to be mapped and cloned were the breast and ovarian cancer susceptibility genes BRCA1 (Miki *et al*, 1994) and BRCA2 (Wooster *et al*, 1995). It is estimated that BRCA1 and BRCA2 are responsible for 5 – 10% of breast cancer cases (King *et al*, 1993; Eeles, 1996) and 10% of ovarian cancer cases (Easton *et al*, 1993; Couch and Weber, 1998; Landis *et al*, 1999; Risch *et al*, 2001). Women with a BRCA1 mutation and multiple cases of BRCA1 mutations in their families can have a lifetime risk in excess of 80% of developing breast cancer, a 40 – 60% chance of developing ovarian cancer and possibly an increased risk of developing colorectal cancer (Ford *et al*, 1994).

As a consequence of increased awareness of genetic issues and the technology to test for mutations there has been demand for genetic assessment services (Evans *et al*, 1994; Campbell *et al*, 1995; Priority Areas Cancer Team, 1998; Ponder, 1999), demand that is likely to increase (Ponder, 1999). However, as cancer mutations have only been isolated in the last decade, clinical trials, cancer registry and observational studies will take years to collect data, establishing long-term costs, and assess the efficacy of surveillance and prophylactic interventions in preventing cancer and prolonging life. In the mean time, researchers have begun to look at these long-term outcomes by means of mathematical modelling. Studies assessing the potential costs and benefits of genetic testing accompanied by interventions for women free of cancer but with a family history of breast and ovarian cancer (Schrag *et al*, 1997; Grann *et al*, 1998, 1999a, 2000, 2002; Tengs *et al*, 1998; Tengs and Berry, 2000) found gains in life years for women opting for bilateral prophylactic mastectomy and/or oophorectomy (Schrag *et al*, 1997; Grann *et al*, 1998, 2000, 2002) or chemoprevention methods such as tamoxifen or raloxifene (not available in the UK) (Grann *et al*, 2000, 2002) when compared with presymptomatic surveillance alone.

In terms of assessing the survival advantages of providing cancer genetic services to women at increased risk of breast cancer the preceding studies have not addressed three important issues. Firstly, despite varying the penetrance estimates (the likelihood of developing cancer), they have confined their analysis to BRCA1/2 mutation carriers only. However, the majority of individuals referred to cancer genetic services due to a family history placing them at increased risk of developing breast cancer do not have BRCA1/2 mutations. Most high-risk and all moderate-risk families have as yet unidentified mutations. Secondly, all women have been assumed to be at high risk of developing both breast and ovarian cancer. However, the Cancer Genetics Service in Wales referral guidelines, which were developed by means of a national multidisciplinary consensus group meeting and GP focus groups, which are subject to ongoing validation, segregate women according to their family history into increased risk of developing breast cancer or breast and ovarian cancer. This has implications both for the type of counselling to be provided and the type of presymptomatic surveillance required. Finally, none of these studies have compared the provision of genetic services with the treatment as usual of no presymptomatic services.

This paper stems from the work conducted by the GenQuest research team who conducted a multimethod evaluation of the Cancer Genetics Service in Wales. The aim of this paper is to compare a service offering genetic testing and presymptomatic surveillance to women at high and moderate risk of developing breast cancer with its predecessor of no service at all. This will be done by means of mathematical modelling, using available epidemiological data and reasonable assumptions.

## MATERIALS AND METHODS

### Model design

**Figure 1:**
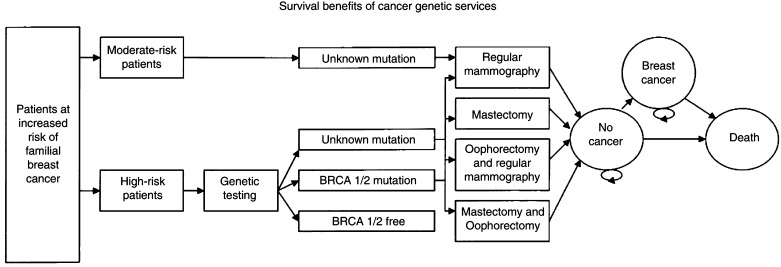
Markov state diagram.

A Markov cohort chain simulation model was developed and run on Lotus 123 to estimate survival and quality-adjusted survival for women at increased risk (high and moderate) of developing breast cancer under the care of genetic services compared to women receiving no such service. Three health states were considered: good health, breast cancer and death (see Figure 1

). Additionally, the impact of regular mammography, bilateral prophylactic mastectomy, prophylactic oophorectomy with regular mammography or prophylactic mastectomy with oophorectomy was accounted for in the simulated cohort receiving genetic assessment services. Women were followed for a maximum 24 years (Markov cycles), starting at 35 years of age and terminating at death or delayed entry into a national screening programme. Transition between health states was assumed to occur annually. Risk estimates of transition from one state to another were transformed into annual transition probabilities by means of the actuarial and the simple cumulative methods (Miller and Homan, 1994).

### Health parameters

Women are defined as being at increased risk of developing breast cancer if they have one of the following four family histories on the same side of the family; one, first-degree relative (sister, mother or daughter) diagnosed with breast cancer at 40 years of age or less, two, first-degree relatives diagnosed with cancer at 60 years of age or less, three, first-or second-degree relatives (grandparent, grandchild, uncle, aunt, nephew, niece or half-sibling) diagnosed with breast cancer at any age or a first-degree relative with bilateral breast cancer. Women at increased risk are segregated into high and moderate risk using CYRILIC software. Women with a heterozygote risk (chance of having a mutation) of less than 25% are at moderate risk while those with a risk of 25% or more are at high risk. An example of a high-risk family history would be a woman with two first-degree relatives diagnosed with breast cancer at 35 years of age. The genetic testing and presymptomatic surveillance strategies modelled in this study are based upon those used by the Cancer Genetics Service in Wales, a national cancer genetics service (see Table 1

**Table 1:** Care pathways

**Genetic status**	**Genetic service**	**No genetic services**
Low risk	Reassured that they are at population risk. Enter national screening programme at 50 years of age.	Enter national screening programme at 50 years of age.
Moderate risk	Phone counselling and regular mammography for women who wish to have presymptomatic surveillance. Annual mammography from 40 to 50 and every 18 months from 50 to 60 years of age. Women enter the national screening programme at 60 years of age.	Enter national screening programme at 50 years of age.
High risk – did not inherit a BRCA1/2 mutation	Face-to-face counselling and genetic testing having found a BRCA1/2 mutation in a cancer-affected relative. Informed that they are at population risk. Enter national screening programme at 50 years of age.	Enter national screening programme at 50 years of age.
High risk – inherited a BRCA1/2 mutation	Face-to-face counselling and genetic testing having found a BRCA1/2 mutation in a cancer-affected relative. Women can then opt to have regular mammography, masteclomy, oophorectomy and regular mammography or mastectomy with oophorectomy. Surveillance is provided in the form of annual mammography from the age of 35–50 years and every 18 months from 50 to 60 years of age. Women enter the national screening programme at 60 years of age.	Enter national screening programme at 50 years of age.
High risk – unknown mutation in family	Face-to-face counselling but no genetic testing as a BRCA1/2 mutation was not found in a cancer-affected relative. Women can then opt to have regular mammography, mastectomy, oophorectomy and regular mammography or mastectomy with oophorectomy. Surveillance is provided in the form of annual mammography from the age of 35–50 years and every 18 months from 50 to 60 years of age. Women enter the national screening programme at 60 years of age.	Enter national screening programme at 50 years of age.

High-risk women that did not inherit a BRCA1/2 mutation and low-risk women are not included in the Markov model. The national screening programme is based upon the UK breast screening programme providing mammography every 3 years from 50 years of age.

). Both the health parameters that were varied (see Table 2

**Table 2:** Base-case probabilities and sensitivity analysis parameter estimates

**Parameters**	**High risk [sensitivity estimates]**	**Moderate risk [sensitivity estimates]**
Mutation prevalence	18% (Stoppa-Lyonnet *et al*, 1997) [7% (Couch *et al*, 1997; Stoppa-Lyonnet *et al*, 1997), 27% (Eccles *et al*, 1998)]	
Mutation penetrance	**BRCA1/2**	25%^a^ [17%, 33%]^c^
	82% (Easton *et al*, 1993) [70%^a^ , 82%]	
	**Unidentified mutation**	
	40%^a^ [35%, 40%]^a^	
Prophylactic mastectomy	5%^c^ [5%^c^, 38%^d^]	0%^c^
Prophylactic oophorectomy	5%^c^ [0%^c^, 14%^d^]	0%^c^
Prophylactic mastectomy and oophorectomy	5%^c^ [0%^c^, 13%^d^]	0%^c^
5 year death rate for genetics service patients	*35 – 54 years* 7.8% – 2 year lead time [3.8% with no lead time, 9.4% with a 2 year lead time]^b^	*35*–*54 years* 7.8% – 2 year lead time [3.8% with no lead time, 9.4% with a 2 year lead time]^b^
	*55 – 59 years* 11.5% – 2 year lead time [6.9% with no lead time, 16.7% with a 2 year lead time]^b^	*55 – 59 years* 11.5%-2 year lead time [6.9% with no lead time, 16.7% with a 2 year lead time]^b^
5 year death rate for nongenetics service patients	*35 – 54 years* 28.4%^b^ [24.4%, 35.1%) – no lead time^b^	*35–54 years* 28.4%^b^ [24.4%, 35.1%] – no lead time^b^
	*55 – 59 years* 34.9% [30.2%, 39.0%] – no lead time^b^	*55 – 59 years* 34.9% [30.2%, 39.0%] – no lead time^b^
Number of Markov cycles/years	24 [15, 24]	19 [9, 19]

a PDP Pharoah (personal communication, 2002).

b H Beer and H Fielder, Breast Test Wales (personal communication, 2002).

c Estimated by this research team.

d Up to 35 – 51% of women at increased risk of developing breast cancer (Hatcher *et al*, 2001; Meijers-Heijboer *et al*, 2003) have been found to have prophylactic mastectomy and 27 – 49% (Meijers-Heijboer *et al*, 2003; Schwartz *et al*, 2003) of women at increased risk of developing ovarian cancer have opted to have prophylactic oophorectomy. As this model deals solely with women with a family history of breast cancer, we estimated that a maximum of 51% of women will have mastectomy (38% mastectomy alone +13% mastectomy and oophorectomy) and 27% will have oophorectomy (14% oophorectomy alone +13% mastectomy and oophorectomy).

) and those held constant were derived from peer review literature, national statistics and consultation with epidemiologists.

#### Constant modelling parameters

Half the offspring of BRCA1/2 mutation carriers will inherit their parents' mutations (US Department of Health & Human Services, 1999). A woman's likelihood of developing breast cancer was reduced by 90% by having bilateral mastectomy (Ziegeler and Kroll, 1991; Hartmann *et al*, 1997, 1999), reduced by 40% by having prophylactic oophorectomy (Grann *et al*, 2002) and reduced by 91% by having both surgeries (Tengs and Berry, 2000). All women dying from breast cancer are assumed to have metastatic cancer for the last 12 months of their life. All-cause mortality for women was assumed to be 0.26% annually (National Statistics, 2001). Of women receiving annual mammography for 10 years or more, 0.01% will die annually due to the radiation from mammography (National Institute of Health Consensus Development Panel, 1997). Assuming that two blood/DNA samples are analysed a false-negative rate for presymptomatic BRCA1/2 tests would be expected to be less than 0.01% (R Butler, personal communication, 2002).

### Quality of life estimates

Life years were converted to quality-adjusted life years (QALYs) by applying preference ratings. For the health states of bilateral prophylactic mastectomy, prophylactic oophorectomy, having both bilateral prophylactic mastectomy and oophorectomy, cancer, metastatic cancer and death, preference ratings were used from a study of women aged 33 – 50 with a family history of breast and ovarian cancer (Grann *et al*, 1999b). The preference ratings were 0.76 (s.d. 0.26) for bilateral prophylactic mastectomy, 0.82 (s.d. 0.27) for prophylactic oophorectomy, 0.73 (s.d. 0.25) for both bilateral prophylactic mastectomy and prophylactic oophorectomy, 0.83 (s.d. 0.27) for cancer, 0.59 (s.d. 0.27) for metastatic cancer and 0.0 for death. For presymptomatic women who had not developed cancer yet and not opted to have a prophylactic surgery, a study of women approaching the Cancer Genetics Service in Wales for genetic assessment was conducted. Mean ratings for the self-rated health status scale of the EuroQol EQ-5D (EuroQol Group, 1990) were transformed to proportions for the 322 women responding at baseline (approaching the service) and 124 responding postrisk assessment. The mean rating of 0.77 was very similar to the findings of Grann *et al* (1999b) of 0.76 (s.d. 0.29).

### Discounting

As the benefits of cancer genetic services occur in the future these benefits will be discounted. Discounting assumes that future benefits are valued less than current benefits and allows comparison of the benefits of programmes with differing time profiles to be compared (Jones, 1997; Torgerson and Raftery, 1999). Quality-adjusted life years were discounted at 1.5% as recommended by the National Institute of Clinical Excellence and the Treasury (personal communication with NICE, 2002).

### Sensitivity analysis

As there is uncertainty about many of the parameters associated with genetic breast cancer (Easton *et al*, 1993, 1995; Ford *et al*, 1995; Couch *et al*, 1997; Serova *et al*, 1997; Stoppa-Lyonnet *et al*, 1997; Struewing *et al*, 1997; Whittemore *et al*, 1997; Eccles *et al*, 1998; Grann *et al*, 1999b), the uncertainty was accounted for by conducting one-way and multi-way sensitivity analysis upon the following model parameters:

- Prevalence of the mutation.
- Penetrance of the mutation.
- Percentage deciding to have bilateral mastectomy.
- The death rate of women receiving genetic services as survival times for screen-detected cancers are artificially inflated by early detection or lead time (Morrison, 1992).
- The death rate of women not receiving genetic services and presymptomatic surveillance.
- Mean age of women entering the care pathways of the genetic service.
- QALY preference ratings.
- Discount rate.

Of all the women receiving mammography in a given year, 0.2% of those with breast cancer will not be detected (H Beer & H Fielder of Breast Test Wales, personal communication, 2002). The impact of missed cancers by mammographic surveillance was assumed to be negligible on the outcome measures of the modelling in the base-case analysis. By conducting sensitivity analysis on the death rates of women receiving and not receiving genetic services the lead time and the efficacy of mammography and surgery are accounted for.

UK population norms for the self-rated health status scores of the EuroQol EQ-5D (Kind *et al*, 1999) were transformed to proportions and used to simulate cancer genetic services increasing, decreasing and causing no change in adverse psychological effects relative to women receiving no genetic services. See Table 3

**Table 3:** QALY parameter estimates

**State**	**Gen. services**	**No gen. services**
*If equal adverse psychological effects (base case)*		
		
Cancer free	0.77	0.77
Prophylactic mastectomy	0.76 (Grann *et al*, 1999b)	—
Prophylactic oophorectomy	0.82 (Grann *et al*, 1999b)	—
Mastectomy and oophorectomy	0.73 (Grann *et al*, 1999b)	—
Breast cancer	0.83 (Grann *et al*, 1999b)	0.83 (Grann *et al*, 1999b)
Metastatic cancer	0.59 (Grann *et al*, 1999b)	0.59 (Grann *et al*, 1999b)
Death	0.0 (Grann *et al*, 1999b)	0.0 (Grann *et al*, 1999b)
		
*If gen. services creates adverse psychological effects*		
		
Cancer free	0.77	0.86 (Serova *et al*, 1997)
Prophylactic mastectomy	0.76 (Grann *et al*, 1999b)	—
Prophylactic oophorectomy	0.82 (Grann *et al*, 1999b)	—
Mastectomy and oophorectomy	0.73 (Grann *et al*, 1999b)	—
Breast cancer	0.83 (Grann *et al*, 1999b)	0.83 (Grann *et al*, 1999b)
Metastatic cancer	0.59 (Grann *et al*, 1999b)	0.59 (Grann *et al*, 1999b)
Death	0.0 (Grann *et al*, 1999b)	0.0 (Grann *et al*, 1999b)
		
*If gen. services reduce adverse psychological effects*		
		
Cancer free	0.77	0.68^a^
Prophylactic mastectomy	0.76 (Grann *et al*, 1999b)	—
Prophylactic oophorectomy	0.82 (Grann *et al*, 1999b)	—
Mastectomy and oophorectomy	0.73 (Grann *et al*, 1999b)	—
Breast cancer	0.83 (Grann *et al*, 1999b)	0.83 (Grann *et al*, 1999b)
Metastatic cancer	0.59 (Grann *et al*, 1999b)	0.59 (Grann *et al*, 1999b)
Death	0.0 (Grann *et al*, 1999b)	0.0 (Grann *et al*, 1999b)

a Estimated by the research team.

for the QALY parameter estimates. The discount rates applied were 0, 1.5 and 6%. All remaining parameter estimates are recorded in Table 2.

## RESULTS

### Survival

The analysis revealed that genetic assessment followed by regular presymptomatic surveillance provided greater life expectancy than no health services, providing between 0.07 and 1.61 mean additional life years (see Table 4

**Table 4:** Mean incremental health outcomes of genetic services compared to no presymptomatic health services

	**Base case**		**Least favourable**		**Most favourable**	
**Risk level and clinical strategy**	**Life years**	**QALYs**	**Life years**	**QALYs**	**Life years**	**QALYs**
High-risk BRCA1/2 carriers						
Genetic services	**1.07**	**0.69**	**0.07**	**0.03**	**1.72**	**0.94**
Surveillance	0.99	0.68	0.06	0.04	1.57	1.05
Mastectomy	1.61	0.61	0.23	−0.06	1.83	0.76
Oophorectomy	1.36	1.67	—	—	1.72	1.91
Mastectomy and oophorectomy	1.61	0.003	—	—	1.83	0.15
						
High-risk carriers of an unidentified mutation						
Genetic services	**0.34**	**0.22**	**0.02**	**0.009**	**0.55**	**0.27**
Surveillance	0.32	0.21	0.02	0.02	0.50	0.33
Mastectomy	0.52	0.05	0.08	−0.11	0.59	0.10
Oophorectomy	0.41	1.21	—	—	0.54	1.29
Mastectomy and oophorectomy	0.52	−0.55	—	—	0.59	−0.51
						
Moderate risk						
Genetic services	**0.07**	**0.05**	**0.00**	**0.00**	**0.2**	**0.11**
Surveillance	0.07	0.05	0.00	0.00	0.2	0.11
						
All at increased risk						
Genetic services	**0.09**	**0.06**	**0.001**	**0.0005**	**0.18**	**0.12**

Life years were undiscounted and QALYs were discounted at 1.5%.

). Mean incremental life expectancy increased for high-risk compared to moderate-risk carriers and for high-risk BRCA1/2 mutation carriers compared to high-risk carriers of unidentified mutations. Prophylactic oophorectomy extended mean life expectancy over surveillance alone by 0.09–0.37 years and over no presymptomatic health services by 0.41 – 1.36 years. Bilateral prophylactic mastectomy or having both bilateral prophylactic mastectomy and oophorectomy extended mean life expectancy over surveillance alone by 0.20–0.62 years and over no presymptomatic health services by 0.52–1.61 years.

### Quality-adjusted survival

Having adjusted survival to take account of quality of life, genetic services were still found to provide additional life years with the exception of women opting to have both bilateral prophylactic mastectomy and oophorectomy. The hierarchy of QALYs gained mirrored those found for unadjusted survival, except for women having both mastectomy and oophorectomy, with most QALYs being found for BRCA1/2 carriers (0.68–1.67 QALYs), followed by high-risk carriers of unidentified mutations (0.05–1.21 QALYs) and finally moderate-risk mutation carriers (0.05 QALYs). In contrast to the findings for unadjusted survival, when quality of life was accounted for the optimal clinical strategy was oophorectomy with regular surveillance (1.21–1.67 QALYs), followed by surveillance (0.21–0.68 QALYs), bilateral prophylactic mastectomy (0.05–0.61 QALYs) and finally having bilateral prophylactic mastectomy with oophorectomy (−0.55–0.003 QALYs). Unlike the alternative clinical strategies, oophorectomy with regular surveillance recorded more incremental QALYs than unadjusted survival. A result also found by Grann *et al* (2000, 2002). Despite providing the greatest incremental survival, mastectomy and having both mastectomy and oophorectomy provided less QALYs than surveillance. In comparison to having no cancer genetic services the clinical strategy of having both a mastectomy and an oophorectomy provided almost no incremental QALYs for high-risk BRCA1/2 mutation carriers (0.003 QALYs) and was inferior for high-risk carriers of unidentified mutations (−0.55 QALYs).

### Sensitivity analysis

Both life years and QALYs were sensitive to all the parameters varied in this study. Multi-way sensitivity analysis revealed that the gain in mean life years from coordinated genetic services compared to no services were at their maximum of 0.18 when the parameter estimates were set at their most favourable (see Tables 4 and 5

**Table 5:** Most and least favourable parameter estimates

	**Genetic services**		**No presymptomatic services**	
**Parameters**	**High risk**	**Moderate risk**	**High risk**	**Moderate risk**
*Most favourable*				
Mutation prevalence	27%	—	27%	—
Mutation penetrance	82% (BRCA1/2) 40% (unidentified mutation)	33%	82% (BRCA1/2) 40% (unidentified mutation)	33%
Prophylactic mastectomy	38%	0%	0%	0%
Prophylactic oophorectomy	14%	0%	0%	0%
Prophylactic mastectomy and oophorectomy	13%	0%	0%	0%
5 year death rate	*35–54 years* 3.8% with no lead time	*35–54 years* 3.8% with no lead time	*35–54 years* 35.1% with no lead time	*35–54 years* 35.1% with no lead lime
	*55–59 years* 6.9% with no lead time	*55–59 years* 6.9% with no lead time	*55–59 years* 39.0% with no lead time	*55–59 years* 39.0% with no lead time
Number of Markov cycles/years	24	19	24	19
*Least favourable*				
Mutation prevalence	7%	—	7%	—
Mutation penetrance	70% (BRCA1/2) 35% (Unidentified mutation)	17%	70% (BRCA1/2) 35% (unidentified mutation)	17%
Prophylactic mastectomy	5%	0%	0%	0%
Prophylactic oophorectomy	0%	0%	0%	0%
Prophylactic mastectomy and oophorectomy	0%	0%	0%	0%
5 year death rate	*35–54 years* 9.4% with a 2 year lead time	*35–54 years* 9.4% with a 2 year lead time	*35–54 years* 24.4% with no lead time	*35–54 years* 24.4% with no lead time
	*55–59 years* 16.7% with a 2 year lead time	*55–59 years* 16.7% with a 2 year lead time	*55–59 years* 30.2% with no lead time	*55–59 years* 30.2% with no lead time
Number of Markov cycles/years	15	9	15	9

). The minimum gain of 0.001 mean life years was obtained for the least favourable parameter estimates (see Tables 4 and 5).

Applying a 0% discount rate to the base-case analysis provided 0.073 QALYs for the average woman attending cancer genetic services. A 6% discount rate however resulted in only an additional 0.036 mean QALYs for these women. Changing the mean age of women in the base-case analysis from 35 to 44 for high-risk women and from 40 to 50 for moderate-risk women resulted in genetic services only extending survival by 0.003 years or 1 day.

Varying QALY weightings for the base-case parameters resulted in substantial change in the mean incremental QALY outcomes. Substituting weightings indicative of reducing adverse psychological effects among presymptomatic women (see Table 3) resulted in genetic services giving a mean incremental QALY of 1.55 compared to no presymptomatic health services. In the event of genetic services increasing adverse psychological effects compared to no health services for presymptomatic women, the no health services option provided 1.43 mean incremental QALYs over genetic services.

## DISCUSSION

The overall gains in survival and QALYs found in this study are low, particularly for high-and moderate-risk carriers of unidentified mutations. However, quality of life was better for women that were under the care of cancer genetic services with the exception of women for who contact with genetic services would induce adverse psychological effects and those women opting to have both prophylactic mastectomy and oophorectomy.

In Table 4, the difference by risk status in the incremental survival for women having presymptomatic care is a result of mutation penetrance and the ability to identify mutation carriers. Greater incremental survival is associated with higher mutation penetrance or risk of developing breast cancer, the greater the risk of death from cancer the greater the potential saving in life years from surveillance and/or a health intervention. In the autosomal dominant mode of transmission, half of the offspring will be at risk for developing the disease. In the case of an identified mutation such as BRCA1 or BRCA2, it is possible to isolate the individuals inheriting the mutation and target them for surveillance. As the high-risk unidentified mutations and moderate-risk mutations have not been isolated yet, it is necessary to extend presymptomatic surveillance to all members of such families, resulting in lower mean survival than would be achieved by targeted use of presymptomatic surveillance for the two patient groups.

Despite the clear advantage of the three prophylactic surgeries over surveillance alone in terms of extending survival, this was not the case when accounting for quality of life. The preference or QALY weightings used in the current study reflect the fact that although mastectomy and mastectomy with oophorectomy may prevent the physical, social, psychological and economic impact of living with cancer, it may also result in altered body image, a sense of reduced femininity and physical discomfort from surgery (Grann *et al*, 1998). There is also much uncertainty confronting women having to make a decision about prophylactic surgery. None of the prophylactic surgeries are 100% effective in preventing cancer, not all mutation carriers will develop cancer and half of the women from families with a known or unidentified mutation will not inherit that mutation. Prophylactic surgery is likely to be most appropriate for women with low concerns about the impact of surgery upon their quality of life or for whom the impact of surgery may be substantial but less severe than having to live with the constant knowledge that they are at increased risk of developing breast cancer.

The low incremental survival found for women who postponed entry into the genetic service was a result of two factors. Firstly, older women have less potential gains in life expectancy and less time under the care of genetic service before entering conventional surveillance at 60 years of age. Secondly, due to lack of data it has been necessary to assume equal sensitivity for the 1–1.5 yearly mammography for patients of the genetics service and three yearly mammography provided to women receiving no presymptomatic health services until they enter the national screening programme at 50 years of age.

In comparison to the findings of Schrag *et al* (1997) and Grann *et al* (1998, 2000, 2002), who recoded incremental survival of between 2.8–5.3 life years for women having mastectomy, 0.8–2.6 years for oophorectomy and 4.3–7.6 years for mastectomy and oophorectomy (Schrag *et al*, 1997; Grann *et al*, 1998, 2000, 2002) compared to women having surveillance alone, the results for BRCA1/2-positive women in the current study appear to be low. There are three main reasons for this discrepancy. Firstly, Grann *et al* ran their models for 50 (Grann *et al*, 1998, 2000) and 70 (Grann *et al*, 2002) cycles compared to the 24 used in the current study. Secondly, Schrag *et al* (1997) and Grann *et al* (1998, 2000, 2002) used age-specific risk estimates of developing cancer based upon the work of Struewing *et al* (1997). These estimates were not utilised in the current study modelling health outcomes for the general public as the Struewing *et al* (1997) work was based upon the Ashkenazi Jewish population, an ethnic group known to have a substantially higher prevalence of BRCA1/2 mutations than other ethnic groups, 1.0–2.5% (Struewing *et al*, 1995; Oddoux *et al*, 1996; Tonin *et al*, 1996) compared to 0.25–0.5% in the general public (Brooks, 1999) and lower penetrance or lifetime risk (Ford *et al*, 1995; Struewing *et al*, 1997). Finally, Schrag *et al* (1997) and Grann *et al* (1998, 2000, 2002) were modelling survival for women with a BRCA1/2 mutation and a family history predisposing them to both breast and ovarian cancer.

It should be noted that chemoprevention methods have also been modelled as presymptomatic interventions for women at increased risk of developing breast and ovarian cancer due to BRCA1/2 mutations. Compared to women having surveillance alone raloxifene provided 2.2 incremental life years (Grann *et al*, 2000), tamoxifen 1.6–1.8 years (Grann *et al*, 2000, 2002) and tamoxifen with oophorectomy 4.6 years (Grann *et al*, 2002). These interventions were not considered in this study as they have not been approved for use in the UK.

There are limitations to the current study which must be noted. Firstly, in the absence of age-specific risk or penetrance estimates of developing breast cancer, it was necessary to base annual penetrance estimates upon lifetime risk estimates. Secondly, the data upon mortality as a result of radiation from mammography are based on estimates for women aged 40–49 years (National Institute of Health Consensus Development Panel, 1997). It may be that the risk varies considerably depending on the age at which mammography commences and the duration of attendance for women with a cancer genetic mutation. Thirdly, mortality estimates are based upon the latest data available from Breast Test Wales. However, this is based upon three yearly mammography for women aged 50–59 who do not have a family history of cancer. Depending upon how rapidly genetic cancer develops these estimates may under-or overestimate the effectiveness of annual mammography from 35/40 to 50 years and 18 monthly from 50 to 60 years for women with a family history of cancer. Fourthly, cancer recurrence has not been accounted for. Fifthly, environmental and lifestyle issues, and changes in these factors following advice and information from the genetic service could not be accounted for. Sixthly, as this study estimates incremental survival a major patient group is excluded from the results of this analysis. This group comprises of individuals from families with a known BRCA1/2 mutation that have not inherited the mutation predisposing them to increased risk of developing cancer and for whom the main outcomes are psychological. Finally, it has been proposed that the wide range of positive and negative outcomes associated with genetic testing such as reduced uncertainty or knowing that a child has not inherited a mutated gene cannot be aggregated into a single measure such as a QALY (Van der Riet *et al*, 1997). In the future, as epidemiology for this patient group improves it will be possible to rectify many of these limitations. Although several of the above limitations would impact upon the differential in survival and QALYs between genetic services and no such services we do not envisage that the overall conclusions of the study would change.

In conclusion, the results of the Markov modelling indicate that providing women at increased risk of developing breast cancer with cancer genetic services will give a small increase in their estimated survival. As long as genetic services do not induce adverse psychological effects they also provide greater quality of life. The greatest survival and QALY benefits were found for women with identified mutations such as BRCA1 or BRCA2. As more cancer predisposing genes are identified, the survival benefits and cost-effectiveness of genetic testing and presymptomatic surveillance will improve for women at increased risk of developing breast cancer. Although prophylactic mastectomy and mastectomy with oophorectomy were found to be the most effective in extending survival, when quality of life was accounted for surveillance alone was a better option and oophorectomy accompanied by regular surveillance was found to be the optimal clinical strategy for the average woman. Delayed entry into coordinated genetic services was found to diminish the average survival and QALY gains for a woman utilising these services.
